# Supplementary material for: Prevalence of Chronic Kidney Disease and Variation of Its Risk Factors by the Regions in Okayama Prefecture
Source: J Pers Med. 2022 Jan 12;12(1):97. doi: 10.3390/jpm12010097 (PMC8781595; doi:10.3390/jpm12010097)
Supplement: Supplementary file 1 [file jpm-12-00097-s001.zip › jpm-1514415-supplementary.pdf]

Supplementary Materials:

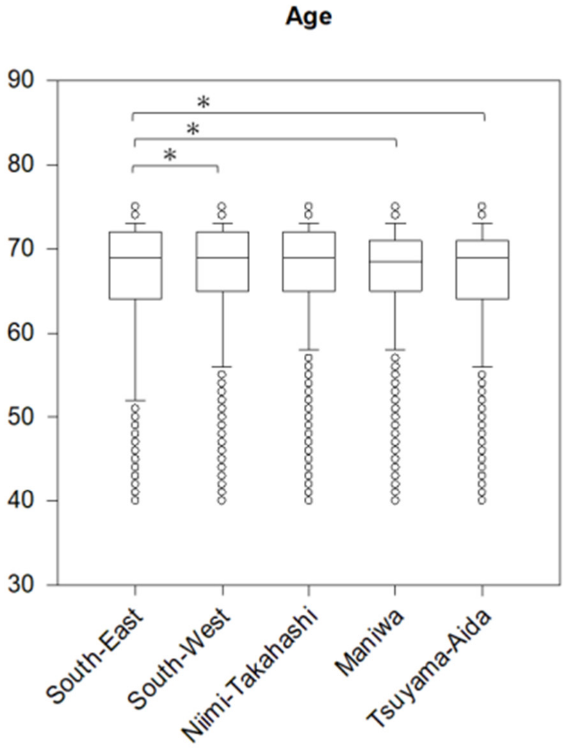

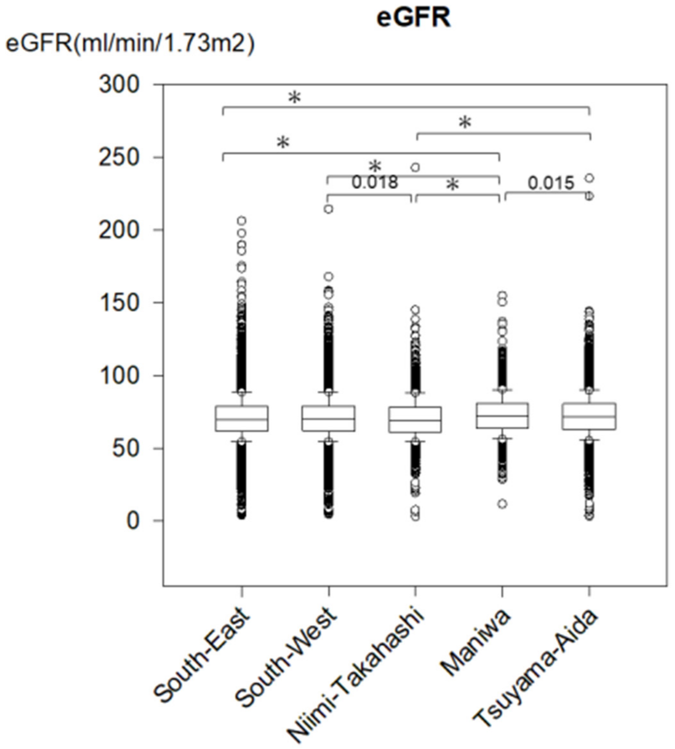

Figure S1. Actual value of age and eGFR. $p < 0.001$

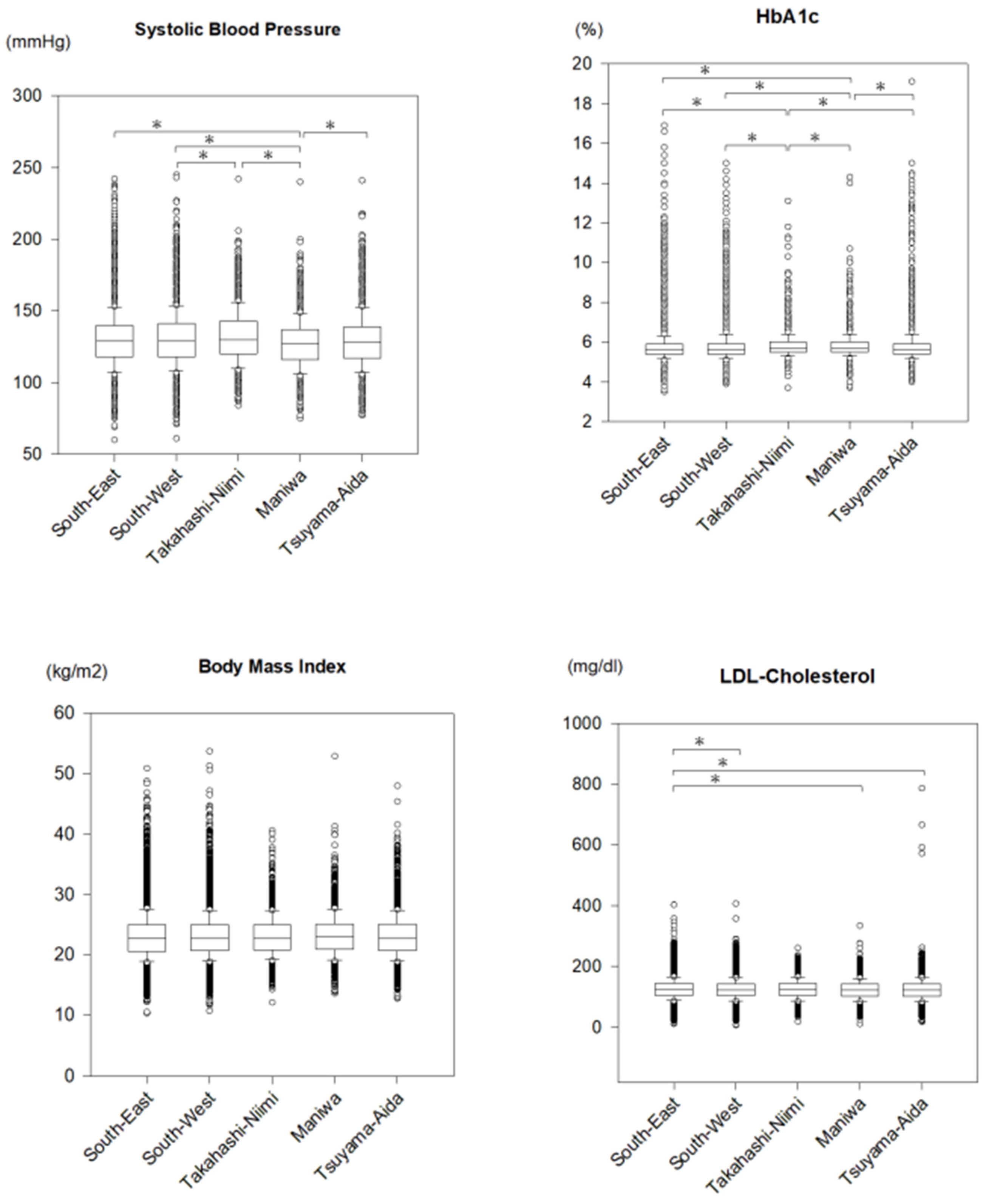

Figure S2. Actual values of systolic blood pressure, HbA1c, Body mass index and LDLcholesterol. $p < 0.001$.
